# Melaminium nitrate–melamine–water (1/1/1)

**DOI:** 10.1107/S1600536810043941

**Published:** 2010-10-31

**Authors:** Farook Adam, Sek Kei Lin, Kasim Mohammed Hello, Madhukar Hemamalini, Hoong-Kun Fun

**Affiliations:** a School of Chemical Sciences, Universiti Sains Malaysia, 11800 USM, Penang, Malaysia; b School of Chemistry, Universiti Sains Malaysia, 11800 USM, Penang, Malaysia; c X-ray Crystallography Unit, School of Physics, Universiti Sains Malaysia, 11800 USM, Penang, Malaysia

## Abstract

In the crystal structure of the title salt, C_3_H_7_N_6_^+^·NO_3_^−^·C_3_H_6_N_6_·H_2_O, the asymmetric unit consists of two neutral melamine (1,3,5-triazine-2,4,6-triamine) mol­ecules, two melaminium cations, two nitrate anions and two solvent water mol­ecules. One of the nitrate anions is disordered over two sets of positions, with a refined occupancy ratio of 0.909 (3):0.091 (3). The cations and neutral mol­ecules are approximately planar, with maximum deviations of 0.018 (2), 0.024 (2), 0.019 (2) and 0.007 (2) Å for each, respectively. In the crystal structure, melaminium cations and netural melamine mol­ecules self-assemble *via* N—H⋯N hydrogen bonds to form a supra­molecular hexa­gonal-shaped motif. In addition, the nitrate anions and water mol­ecules are connected by N—H⋯O hydrogen bonds to form a three-dimensional network.

## Related literature

For applications of melamine, see: Rima *et al.* (2008 ▶); Cook *et al.* (2005 ▶); Ramos Silva *et al.* (2008 ▶). For related structures, see: Debrus *et al.* (2007 ▶); Zhao & Shi (2010 ▶); Marchewka & Pietraszko (2003 ▶); Marchewka (2002 ▶). For applications of hydrogen bonding, see: Aghabozorg *et al.* (2008 ▶).
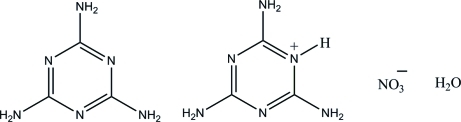


## Experimental

### 

#### Crystal data

C_3_H_7_N_6_^+^·NO_3_^−^·C_3_H_6_N_6_·H_2_O
*M* *_r_* = 333.31
Triclinic, 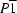

*a* = 7.7759 (1) Å
*b* = 9.0035 (1) Å
*c* = 19.4573 (3) Å
α = 96.182 (1)°
β = 90.854 (1)°
γ = 99.828 (1)°
*V* = 1333.64 (3) Å^3^
*Z* = 4
Mo *K*α radiation
μ = 0.14 mm^−1^
*T* = 296 K
0.21 × 0.14 × 0.09 mm

#### Data collection

Bruker SMART APEXII CCD area-detector diffractometer
Absorption correction: multi-scan (*SADABS*; Bruker, 2009 ▶) *T*_min_ = 0.972, *T*_max_ = 0.987
22196 measured reflections
5160 independent reflections
3689 reflections with *I* > 2σ(*I*)
*R*_int_ = 0.042

#### Refinement

*R*[*F* ^2^ > 2σ(*F* ^2^)] = 0.047
*wR*(*F* ^2^) = 0.123
*S* = 1.03
5160 reflections
543 parameters
All H-atom parameters refined
Δρ_max_ = 0.21 e Å^−3^
Δρ_min_ = −0.45 e Å^−3^

### 

Data collection: *APEX2* (Bruker, 2009 ▶); cell refinement: *SAINT* (Bruker, 2009 ▶); data reduction: *SAINT*; program(s) used to solve structure: *SHELXTL* (Sheldrick, 2008 ▶); program(s) used to refine structure: *SHELXTL*; molecular graphics: *SHELXTL* and *PLATON* (Spek, 2009 ▶); software used to prepare material for publication: *SHELXTL* and *PLATON* (Spek, 2009 ▶).

## Supplementary Material

Crystal structure: contains datablocks global, I. DOI: 10.1107/S1600536810043941/lh5157sup1.cif

Structure factors: contains datablocks I. DOI: 10.1107/S1600536810043941/lh5157Isup2.hkl

Additional supplementary materials:  crystallographic information; 3D view; checkCIF report

## Figures and Tables

**Table 1:** Hydrogen-bond geometry (Å, °)

*D*—H⋯*A*	*D*—H	H⋯*A*	*D*⋯*A*	*D*—H⋯*A*
N4*A*—H1*A*⋯O2*W*^i^	0.92 (2)	2.05 (2)	2.965 (2)	174.2 (17)
N4*B*—H1*B*⋯N1*D*	0.85 (2)	2.18 (2)	3.025 (2)	174.1 (18)
N4*D*—H1*D*⋯O3*AA*	0.88 (2)	2.27 (2)	3.077 (2)	153 (2)
N1*A*—H1N⋯O1*W*^ii^	0.91 (2)	1.89 (2)	2.771 (2)	165 (2)
N4*A*—H2*A*⋯O1*B*^i^	0.96 (3)	2.03 (3)	2.838 (2)	141 (2)
N4*B*—H2*B*⋯O3*AA*	0.93 (3)	2.05 (2)	2.810 (2)	138.8 (18)
N4*C*—H2*C*⋯N2*D*^iii^	0.88 (3)	2.07 (3)	2.945 (2)	176 (2)
N4*D*—H2*D*⋯N2*C*^iii^	0.84 (2)	2.23 (2)	3.069 (2)	172 (2)
N1*C*—H2N⋯O3*B*	0.93 (2)	1.91 (2)	2.836 (2)	177 (2)
O2*W*—H1*W*2⋯N1*D*	0.86 (3)	2.04 (3)	2.899 (2)	174 (3)
N5*A*—H3*A*⋯N2*B*	0.88 (3)	2.20 (3)	3.062 (2)	165 (2)
N5*B*—H3*B*⋯N2*A*	0.82 (3)	2.30 (3)	3.119 (2)	175 (2)
N5*C*—H3*C*⋯O2*AA*^iii^	0.89 (2)	2.06 (2)	2.799 (2)	140 (2)
N5*D*—H3*D*⋯O3*AA*^iv^	0.86 (2)	2.53 (2)	3.232 (3)	140 (2)
N5*A*—H4*A*⋯O1*AA*	0.86 (2)	2.11 (2)	2.963 (2)	171 (2)
N5*B*—H4*B*⋯O2*B*^i^	0.86 (2)	2.20 (2)	3.045 (2)	167 (2)
N5*C*—H4*C*⋯N3*D*^v^	0.89 (3)	2.12 (3)	2.996 (2)	173 (2)
N5*D*—H4*D*⋯N3*C*^v^	0.81 (3)	2.30 (3)	3.105 (3)	173 (2)
N6*A*—H5*A*⋯N3*B*^vi^	0.80 (2)	2.13 (2)	2.926 (2)	175.9 (19)
N6*B*—H5*B*⋯N3*A*^vii^	0.84 (2)	2.18 (2)	3.020 (2)	177 (2)
N6*C*—H5*C*⋯O2*W*	0.93 (3)	2.00 (3)	2.849 (2)	151 (2)
N6*D*—H5*D*⋯O2*AA*^vii^	0.93 (3)	2.22 (3)	3.126 (2)	167 (2)
N6*A*—H6*A*⋯O1*W*^ii^	0.90 (2)	2.55 (2)	3.262 (2)	137.2 (15)
N6*A*—H6*A*⋯O2*B*^viii^	0.90 (2)	2.14 (2)	2.841 (2)	135.2 (17)
N6*B*—H6*B*⋯O1*AA*^vii^	0.89 (2)	2.17 (3)	2.806 (2)	128 (2)
N6*C*—H6*C*⋯O1*B*	0.90 (2)	1.97 (2)	2.868 (2)	174.6 (18)
N6*D*—H6*D*⋯N1*B*	0.91 (2)	2.18 (2)	3.088 (2)	172 (2)

Symmetry codes: (i) 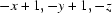; (ii) 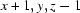; (iii) 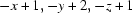; (iv) 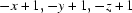; (v) 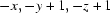; (vi) 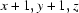; (vii) 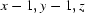; (viii) 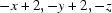.

**Fig. 1.**
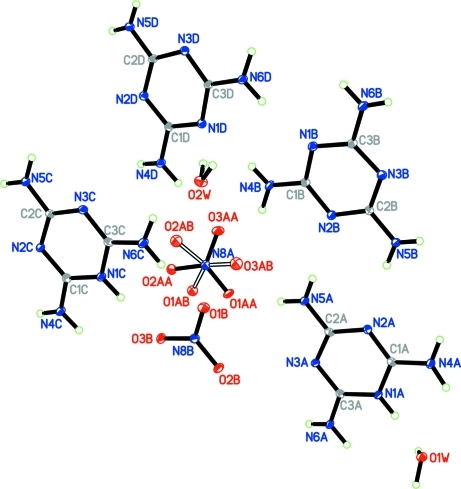
The asymmetric unit of the title compound, showing 30% probability displacement ellipsoids and the atom-numbering scheme. Open bonds represents disorder components.

**Fig. 2.**
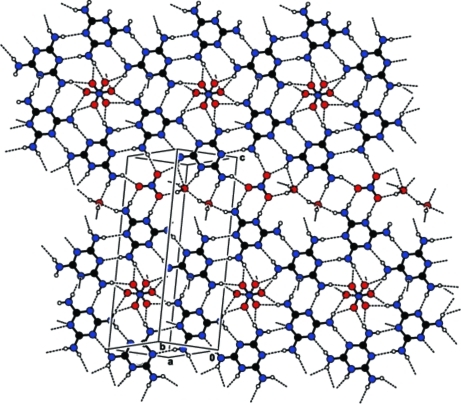
The crystal packing of the title compound, showing the hydrogen-bonded (dashed lines) network.

## supplementary crystallographic information

### Comment

1,3,5-triazine-2,4,6-triamine is an organic base also known as melamine. Melamine is very widely used in several industries, such as the production of melamine foam in polymeric cleaning (Rima *et al.*, 2008) and also as a chemical intermediate in amino resin and plastics manufacturing (Cook *et al.*, 2005). Melamine can be a proton acceptor and will form 2,4,6-triamino-1,3,5-triazine-1-ium (Ramos Silva *et al.*, 2008). Recently many melaminium complexes in crystalline form has been reported, such as melaminium-bis(trichloroacetate) monohydrate (Debrus *et al.*, 2007), melaminium iodide monohydrate (Zhao & Shi, 2010) and melaminium citrate (Marchewka & Pietraszko, 2003). Melaminium salt crystals have shown interesting properties like nonlinear optical behaviour (Marchewka, 2002). In the formation of melaminium salt crystals, molecules are bound to each other via hydrogen bonds. Hydrogen bonding plays an important role in the catalytic, biochemical activities and also in supramolecular chemistry and crystal engineering (Aghabozorg *et al.*, 2008). Here, we report the crystal structure of a melaminium salt. This crystal was obtained as a by-product during our attempt to form crown complexes with melamine.

The asymmetric unit of the title compound consists of two crystallographically independent protonated melaminium cations (A & C), two nitrate anions (A & B), two neutral melamine molecules (B & D) and two water molecules (Fig. 1). One of the nitrate anion is disordered over two sets of position, with refined occupancy ratios of 0.909 (3):0.091 (3). The protonated and neutral melamine molecules are essentially planar, with a maximum deviation of 0.018 (2) Å for atom C2A (molecule A), 0.024 (2) Å for atom C2C (molecule C), 0.019 (2) Å for atom C2B (molecule B) and 0.007 (2) Å for atom C2D (molecule D).

In the crystal structure (Fig.2), the protonated melaminium cations and the neutral melamine molecules self-assemble via N—H···N hydrogen bonds to form a supramolecular hexagonal motif. Furthermore, the nitrate anions and water molecules are connected by N—H···O (Table 1) hydrogen bonds to form a three-dimensional network.

### Experimental

0.36 g (2.856 mmol) of melamine, 0.50 g (2.856 mmol) of 1,4-bis(chloromethyl)benzene and 1.0 ml triethylamine were added into 40 mL acetonitrile and refluxed for 72 hours at 348 K. The white precipitate was collected by simple filtration and dried at 373 K for 24 hours. About 0.5 g of the white precipitate was dissolved in 10 mL distilled water followed by 0.5 g (1.718 mmol) of cobalt(II) nitrate. The pH was adjusted to 7.0 by a few drops of 1.0 M sodium hydroxide. The mixture was stirred for 2 h and then filtered off. The resulting mixture was kept at room temperature for recrystallization. Recrystallization was carried out twice by using distilled water to get the pure crystal.

### Refinement

All the H atoms were located in a difference Fourier map and allowed to refine freely [N—H = 0.80 (2)–0.96 (2) Å and O—H = 0.85 (4)–0.96 (3) Å]. One of the nitrate anion is disordered over two sets of positions, with refined occupancy ratios of 0.909 (3):0.091 (3).

### Crystal data

**Table:**

C_3_H_7_N_6_^+^·NO_3_^−^·C_3_H_6_N_6_·H_2_O	*Z* = 4
*M**_r_* = 333.31	*F*(000) = 696
Triclinic, *P*1	*D*_x_ = 1.660 Mg m^−^^3^
Hall symbol: -P 1	Mo *K*α radiation, λ = 0.71073 Å
*a* = 7.7759 (1) Å	Cell parameters from 3833 reflections
*b* = 9.0035 (1) Å	θ = 2.3–29.8°
*c* = 19.4573 (3) Å	µ = 0.14 mm^−^^1^
α = 96.182 (1)°	*T* = 296 K
β = 90.854 (1)°	Block, purple
γ = 99.828 (1)°	0.21 × 0.14 × 0.09 mm
*V* = 1333.64 (3) Å^3^	

### Data collection

**Table:**

Bruker SMART APEXII CCD area-detector diffractometer	5160 independent reflections
Radiation source: fine-focus sealed tube	3689 reflections with *I* > 2σ(*I*)
graphite	*R*_int_ = 0.042
φ and ω scans	θ_max_ = 26.0°, θ_min_ = 2.1°
Absorption correction: multi-scan (*SADABS*; Bruker, 2009)	*h* = −8→9
*T*_min_ = 0.972, *T*_max_ = 0.987	*k* = −11→11
22196 measured reflections	*l* = −23→23

### Refinement

**Table:**

Refinement on *F*^2^	Primary atom site location: structure-invariant direct methods
Least-squares matrix: full	Secondary atom site location: difference Fourier map
*R*[*F*^2^ > 2σ(*F*^2^)] = 0.047	Hydrogen site location: inferred from neighbouring sites
*wR*(*F*^2^) = 0.123	All H-atom parameters refined
*S* = 1.03	*w* = 1/[σ^2^(*F*_o_^2^) + (0.0625*P*)^2^ + 0.2158*P*] where *P* = (*F*_o_^2^ + 2*F*_c_^2^)/3
5160 reflections	(Δ/σ)_max_ < 0.001
543 parameters	Δρ_max_ = 0.21 e Å^−^^3^
0 restraints	Δρ_min_ = −0.45 e Å^−^^3^

### Special details

**Table:**

Geometry. All esds (except the esd in the dihedral angle between two l.s. planes) are estimated using the full covariance matrix. The cell esds are taken into account individually in the estimation of esds in distances, angles and torsion angles; correlations between esds in cell parameters are only used when they are defined by crystal symmetry. An approximate (isotropic) treatment of cell esds is used for estimating esds involving l.s. planes.
Refinement. Refinement of F^2^ against ALL reflections. The weighted R-factor wR and goodness of fit S are based on F^2^, conventional R-factors R are based on F, with F set to zero for negative F^2^. The threshold expression of F^2^ > 2sigma(F^2^) is used only for calculating R-factors(gt) etc. and is not relevant to the choice of reflections for refinement. R-factors based on F^2^ are statistically about twice as large as those based on F, and R- factors based on ALL data will be even larger.

### Fractional atomic coordinates and isotropic or equivalent isotropic displacement parameters (Å^2^)

**Table:**

	*x*	*y*	*z*	*U*_iso_*/*U*_eq_	Occ. (<1)
N1A	0.9356 (2)	0.62933 (18)	−0.08016 (9)	0.0142 (4)	
N2A	0.7288 (2)	0.48794 (18)	−0.01488 (8)	0.0149 (4)	
N3A	0.9137 (2)	0.71794 (18)	0.03718 (8)	0.0139 (4)	
N4A	0.7628 (2)	0.4097 (2)	−0.12989 (9)	0.0176 (4)	
N5A	0.7043 (2)	0.5825 (2)	0.09767 (9)	0.0172 (4)	
N6A	1.1157 (2)	0.8451 (2)	−0.03080 (10)	0.0165 (4)	
C1A	0.8069 (2)	0.5077 (2)	−0.07424 (10)	0.0139 (4)	
C2A	0.7836 (2)	0.5972 (2)	0.03848 (10)	0.0145 (4)	
C3A	0.9888 (2)	0.7321 (2)	−0.02329 (10)	0.0136 (4)	
N1B	0.2450 (2)	0.17275 (18)	0.20073 (8)	0.0157 (4)	
N2B	0.4686 (2)	0.29201 (18)	0.13081 (8)	0.0149 (4)	
N3B	0.2675 (2)	0.07228 (18)	0.08247 (8)	0.0152 (4)	
N4B	0.4452 (2)	0.3774 (2)	0.24538 (9)	0.0183 (4)	
N5B	0.4750 (2)	0.1976 (2)	0.01677 (9)	0.0160 (4)	
N6B	0.0627 (2)	−0.0409 (2)	0.15269 (10)	0.0196 (4)	
C1B	0.3839 (2)	0.2789 (2)	0.19097 (10)	0.0148 (4)	
C2B	0.4012 (2)	0.1864 (2)	0.07766 (10)	0.0129 (4)	
C3B	0.1951 (2)	0.0700 (2)	0.14507 (10)	0.0148 (4)	
N1C	0.4424 (2)	1.05878 (19)	0.34890 (9)	0.0170 (4)	
N2C	0.4197 (2)	1.15377 (18)	0.46499 (8)	0.0143 (4)	
N3C	0.2251 (2)	0.92779 (18)	0.41389 (8)	0.0158 (4)	
N4C	0.6243 (2)	1.2761 (2)	0.39577 (10)	0.0182 (4)	
N5C	0.2192 (2)	1.0143 (2)	0.52802 (9)	0.0155 (4)	
N6C	0.2541 (3)	0.8500 (2)	0.29859 (9)	0.0221 (4)	
C1C	0.4950 (2)	1.1634 (2)	0.40435 (10)	0.0149 (4)	
C2C	0.2901 (2)	1.0327 (2)	0.46781 (10)	0.0140 (4)	
C3C	0.3043 (3)	0.9436 (2)	0.35478 (10)	0.0162 (4)	
N1D	0.2208 (2)	0.40060 (18)	0.37041 (8)	0.0155 (4)	
N2D	0.2450 (2)	0.48189 (18)	0.49238 (8)	0.0149 (4)	
N3D	0.0365 (2)	0.26250 (18)	0.44780 (8)	0.0145 (4)	
N4D	0.4156 (2)	0.6117 (2)	0.41573 (10)	0.0180 (4)	
N5D	0.0681 (3)	0.3419 (2)	0.56411 (9)	0.0174 (4)	
N6D	0.0204 (2)	0.1911 (2)	0.32993 (9)	0.0180 (4)	
C1D	0.2918 (2)	0.4954 (2)	0.42667 (10)	0.0139 (4)	
C2D	0.1182 (2)	0.3630 (2)	0.49989 (10)	0.0142 (4)	
C3D	0.0951 (3)	0.2865 (2)	0.38480 (10)	0.0137 (4)	
N8A	0.7624 (2)	0.76433 (19)	0.28672 (9)	0.0178 (4)*	
O1AA	0.8234 (2)	0.77664 (18)	0.22811 (8)	0.0211 (4)	0.909 (3)
O2AA	0.7867 (2)	0.87285 (18)	0.33240 (8)	0.0293 (5)	0.909 (3)
O3AA	0.6809 (2)	0.63698 (18)	0.30001 (8)	0.0305 (5)	0.909 (3)
O1AB	0.887 (2)	0.8747 (18)	0.2933 (8)	0.021 (4)*	0.091 (3)
O2AB	0.649 (2)	0.7781 (19)	0.3371 (8)	0.026 (5)*	0.091 (3)
O3AB	0.721 (2)	0.673 (2)	0.2387 (9)	0.029 (5)*	0.091 (3)
N8B	0.5925 (2)	0.96755 (18)	0.17530 (8)	0.0163 (4)	
O1B	0.46913 (19)	0.86023 (16)	0.18015 (7)	0.0240 (4)	
O2B	0.66849 (18)	0.98031 (16)	0.11995 (7)	0.0200 (3)	
O3B	0.64020 (18)	1.06308 (15)	0.22762 (7)	0.0191 (3)	
O1W	0.1736 (2)	0.67667 (17)	0.81644 (8)	0.0188 (3)	
O2W	0.0718 (2)	0.54790 (17)	0.26433 (8)	0.0217 (4)	
H1N	0.997 (3)	0.639 (3)	−0.1192 (12)	0.028 (6)*	
H2N	0.504 (3)	1.061 (3)	0.3083 (12)	0.032 (7)*	
H1A	0.816 (3)	0.430 (2)	−0.1706 (12)	0.023 (6)*	
H2A	0.668 (3)	0.328 (3)	−0.1251 (13)	0.044 (8)*	
H3A	0.622 (3)	0.504 (3)	0.1019 (11)	0.022 (6)*	
H4A	0.739 (3)	0.648 (3)	0.1329 (11)	0.019 (6)*	
H5A	1.154 (3)	0.905 (3)	0.0016 (12)	0.020 (6)*	
H6A	1.157 (3)	0.853 (2)	−0.0732 (12)	0.020 (6)*	
H1B	0.382 (3)	0.377 (2)	0.2808 (11)	0.018 (6)*	
H2B	0.531 (3)	0.460 (3)	0.2400 (11)	0.030 (7)*	
H3B	0.543 (3)	0.276 (3)	0.0113 (11)	0.022 (6)*	
H4B	0.426 (3)	0.137 (3)	−0.0177 (12)	0.025 (6)*	
H5B	0.021 (3)	−0.106 (3)	0.1195 (12)	0.023 (6)*	
H6B	0.014 (3)	−0.044 (3)	0.1938 (12)	0.027 (7)*	
H1C	0.676 (3)	1.277 (3)	0.3554 (13)	0.030 (7)*	
H2C	0.668 (3)	1.346 (3)	0.4295 (13)	0.033 (7)*	
H3C	0.259 (3)	1.082 (3)	0.5641 (12)	0.025 (6)*	
H4C	0.138 (3)	0.934 (3)	0.5318 (12)	0.032 (7)*	
H5C	0.172 (4)	0.762 (3)	0.3000 (13)	0.047 (8)*	
H6C	0.318 (3)	0.858 (2)	0.2606 (11)	0.016 (6)*	
H1D	0.458 (3)	0.616 (2)	0.3741 (11)	0.014 (6)*	
H2D	0.459 (3)	0.669 (3)	0.4510 (12)	0.024 (6)*	
H3D	0.129 (3)	0.396 (3)	0.5980 (12)	0.030 (7)*	
H4D	−0.005 (3)	0.268 (3)	0.5669 (11)	0.026 (7)*	
H5D	−0.051 (3)	0.102 (3)	0.3382 (12)	0.034 (7)*	
H6D	0.082 (3)	0.194 (3)	0.2905 (12)	0.027 (6)*	
H1W1	0.203 (4)	0.765 (3)	0.7943 (14)	0.054 (9)*	
H2W1	0.270 (4)	0.673 (4)	0.8371 (17)	0.084 (12)*	
H1W2	0.116 (4)	0.499 (3)	0.2936 (15)	0.055 (9)*	
H2W2	−0.034 (4)	0.480 (3)	0.2477 (13)	0.042 (7)*	

### Atomic displacement parameters (Å^2^)

**Table:**

	*U*^11^	*U*^22^	*U*^33^	*U*^12^	*U*^13^	*U*^23^
N1A	0.0151 (9)	0.0154 (9)	0.0106 (9)	−0.0008 (7)	0.0026 (7)	0.0005 (7)
N2A	0.0170 (9)	0.0132 (9)	0.0134 (9)	0.0003 (7)	0.0005 (7)	0.0004 (7)
N3A	0.0148 (9)	0.0140 (9)	0.0118 (9)	−0.0006 (7)	0.0013 (7)	0.0015 (7)
N4A	0.0202 (10)	0.0178 (10)	0.0128 (9)	−0.0014 (8)	0.0022 (8)	−0.0009 (7)
N5A	0.0201 (10)	0.0155 (10)	0.0130 (9)	−0.0047 (8)	0.0032 (8)	−0.0005 (8)
N6A	0.0190 (10)	0.0168 (9)	0.0104 (10)	−0.0043 (7)	0.0027 (8)	−0.0021 (8)
C1A	0.0125 (10)	0.0135 (10)	0.0155 (11)	0.0017 (8)	−0.0004 (8)	0.0017 (8)
C2A	0.0137 (10)	0.0131 (10)	0.0171 (11)	0.0023 (8)	−0.0003 (8)	0.0038 (8)
C3A	0.0133 (10)	0.0128 (10)	0.0153 (10)	0.0038 (8)	−0.0014 (8)	0.0016 (8)
N1B	0.0150 (9)	0.0172 (9)	0.0137 (9)	−0.0002 (7)	0.0011 (7)	0.0006 (7)
N2B	0.0151 (9)	0.0156 (9)	0.0130 (9)	0.0005 (7)	0.0013 (7)	0.0007 (7)
N3B	0.0151 (9)	0.0157 (9)	0.0134 (9)	−0.0007 (7)	−0.0004 (7)	0.0010 (7)
N4B	0.0180 (9)	0.0215 (10)	0.0116 (9)	−0.0046 (8)	0.0049 (8)	−0.0027 (8)
N5B	0.0172 (9)	0.0156 (10)	0.0122 (9)	−0.0041 (8)	0.0016 (7)	−0.0003 (8)
N6B	0.0201 (10)	0.0228 (10)	0.0118 (10)	−0.0067 (8)	0.0027 (8)	−0.0011 (8)
C1B	0.0126 (10)	0.0156 (10)	0.0157 (11)	0.0005 (8)	0.0004 (8)	0.0030 (8)
C2B	0.0124 (10)	0.0135 (10)	0.0133 (10)	0.0031 (8)	−0.0012 (8)	0.0024 (8)
C3B	0.0131 (10)	0.0173 (11)	0.0139 (10)	0.0011 (8)	0.0001 (8)	0.0029 (8)
N1C	0.0196 (9)	0.0160 (9)	0.0131 (9)	−0.0026 (7)	0.0029 (7)	−0.0004 (7)
N2C	0.0149 (9)	0.0149 (9)	0.0127 (9)	0.0015 (7)	0.0021 (7)	0.0009 (7)
N3C	0.0189 (9)	0.0136 (9)	0.0134 (9)	−0.0011 (7)	0.0013 (7)	0.0012 (7)
N4C	0.0205 (10)	0.0179 (10)	0.0131 (10)	−0.0037 (8)	0.0036 (8)	−0.0008 (8)
N5C	0.0179 (9)	0.0137 (9)	0.0129 (9)	−0.0021 (8)	0.0019 (7)	−0.0008 (7)
N6C	0.0271 (11)	0.0206 (10)	0.0142 (10)	−0.0064 (8)	0.0055 (8)	−0.0020 (8)
C1C	0.0142 (10)	0.0145 (10)	0.0163 (11)	0.0037 (8)	−0.0006 (8)	0.0014 (8)
C2C	0.0144 (10)	0.0127 (10)	0.0152 (10)	0.0028 (8)	0.0001 (8)	0.0021 (8)
C3C	0.0169 (10)	0.0137 (10)	0.0172 (11)	0.0010 (8)	0.0011 (8)	0.0016 (8)
N1D	0.0168 (9)	0.0140 (9)	0.0139 (9)	−0.0012 (7)	0.0007 (7)	0.0001 (7)
N2D	0.0164 (9)	0.0150 (9)	0.0130 (9)	0.0019 (7)	0.0010 (7)	0.0015 (7)
N3D	0.0162 (9)	0.0142 (9)	0.0119 (9)	0.0000 (7)	0.0005 (7)	0.0004 (7)
N4D	0.0228 (10)	0.0174 (10)	0.0106 (10)	−0.0044 (8)	0.0022 (8)	−0.0007 (8)
N5D	0.0221 (10)	0.0152 (10)	0.0118 (9)	−0.0045 (8)	0.0014 (8)	−0.0005 (8)
N6D	0.0211 (10)	0.0182 (10)	0.0118 (9)	−0.0040 (8)	0.0023 (8)	−0.0003 (7)
C1D	0.0145 (10)	0.0132 (10)	0.0151 (10)	0.0059 (8)	0.0003 (8)	0.0009 (8)
C2D	0.0151 (10)	0.0147 (10)	0.0137 (10)	0.0037 (8)	0.0015 (8)	0.0037 (8)
C3D	0.0152 (10)	0.0143 (10)	0.0123 (10)	0.0040 (8)	0.0005 (8)	0.0029 (8)
O1AA	0.0246 (9)	0.0249 (10)	0.0128 (8)	0.0000 (7)	0.0057 (7)	0.0038 (7)
O2AA	0.0517 (13)	0.0175 (9)	0.0141 (9)	−0.0037 (8)	0.0020 (8)	−0.0033 (7)
O3AA	0.0362 (11)	0.0232 (10)	0.0240 (10)	−0.0163 (8)	0.0075 (8)	0.0003 (7)
N8B	0.0170 (9)	0.0162 (9)	0.0150 (9)	0.0011 (7)	0.0005 (7)	0.0020 (7)
O1B	0.0221 (8)	0.0201 (8)	0.0247 (8)	−0.0096 (6)	0.0060 (7)	−0.0002 (6)
O2B	0.0226 (8)	0.0227 (8)	0.0130 (7)	−0.0007 (6)	0.0058 (6)	0.0013 (6)
O3B	0.0232 (8)	0.0179 (8)	0.0132 (7)	−0.0020 (6)	0.0019 (6)	−0.0028 (6)
O1W	0.0165 (8)	0.0196 (8)	0.0191 (8)	−0.0011 (6)	0.0027 (7)	0.0031 (6)
O2W	0.0239 (9)	0.0194 (8)	0.0204 (8)	−0.0015 (7)	−0.0047 (7)	0.0048 (7)

### Geometric parameters (Å, °)

**Table:**

N1A—C1A	1.368 (2)	N4C—C1C	1.327 (3)
N1A—C3A	1.374 (2)	N4C—H1C	0.89 (2)
N1A—H1N	0.91 (2)	N4C—H2C	0.88 (2)
N2A—C1A	1.328 (2)	N5C—C2C	1.317 (2)
N2A—C2A	1.361 (2)	N5C—H3C	0.90 (2)
N3A—C3A	1.330 (2)	N5C—H4C	0.89 (3)
N3A—C2A	1.355 (2)	N6C—C3C	1.317 (3)
N4A—C1A	1.322 (2)	N6C—H5C	0.93 (3)
N4A—H1A	0.92 (2)	N6C—H6C	0.90 (2)
N4A—H2A	0.96 (3)	N1D—C3D	1.348 (2)
N5A—C2A	1.323 (3)	N1D—C1D	1.361 (2)
N5A—H3A	0.88 (2)	N2D—C1D	1.347 (2)
N5A—H4A	0.86 (2)	N2D—C2D	1.348 (2)
N6A—C3A	1.313 (2)	N3D—C3D	1.341 (2)
N6A—H5A	0.80 (2)	N3D—C2D	1.356 (2)
N6A—H6A	0.89 (2)	N4D—C1D	1.333 (3)
N1B—C1B	1.345 (2)	N4D—H1D	0.88 (2)
N1B—C3B	1.352 (2)	N4D—H2D	0.84 (2)
N2B—C1B	1.358 (2)	N5D—C2D	1.338 (3)
N2B—C2B	1.360 (2)	N5D—H3D	0.86 (2)
N3B—C2B	1.343 (2)	N5D—H4D	0.81 (2)
N3B—C3B	1.350 (2)	N6D—C3D	1.352 (2)
N4B—C1B	1.332 (2)	N6D—H5D	0.93 (2)
N4B—H1B	0.85 (2)	N6D—H6D	0.91 (2)
N4B—H2B	0.93 (2)	N8A—O3AB	1.180 (17)
N5B—C2B	1.330 (2)	N8A—O2AA	1.235 (2)
N5B—H3B	0.82 (2)	N8A—O1AA	1.250 (2)
N5B—H4B	0.86 (2)	N8A—O1AB	1.257 (16)
N6B—C3B	1.328 (3)	N8A—O3AA	1.266 (2)
N6B—H5B	0.85 (2)	N8A—O2AB	1.340 (16)
N6B—H6B	0.89 (2)	N8B—O2B	1.243 (2)
N1C—C1C	1.362 (2)	N8B—O1B	1.253 (2)
N1C—C3C	1.375 (3)	N8B—O3B	1.267 (2)
N1C—H2N	0.93 (2)	O1W—H1W1	0.94 (3)
N2C—C1C	1.330 (2)	O1W—H2W1	0.85 (4)
N2C—C2C	1.359 (2)	O2W—H1W2	0.86 (3)
N3C—C3C	1.322 (2)	O2W—H2W2	0.96 (3)
N3C—C2C	1.362 (2)		
			
C1A—N1A—C3A	119.68 (17)	H3C—N5C—H4C	123 (2)
C1A—N1A—H1N	122.0 (14)	C3C—N6C—H5C	121.6 (16)
C3A—N1A—H1N	117.9 (14)	C3C—N6C—H6C	119.1 (13)
C1A—N2A—C2A	115.58 (16)	H5C—N6C—H6C	118 (2)
C3A—N3A—C2A	115.82 (16)	N4C—C1C—N2C	120.93 (18)
C1A—N4A—H1A	118.4 (13)	N4C—C1C—N1C	117.64 (18)
C1A—N4A—H2A	116.2 (15)	N2C—C1C—N1C	121.43 (18)
H1A—N4A—H2A	125 (2)	N5C—C2C—N2C	117.48 (17)
C2A—N5A—H3A	120.6 (14)	N5C—C2C—N3C	116.36 (18)
C2A—N5A—H4A	118.9 (14)	N2C—C2C—N3C	126.15 (17)
H3A—N5A—H4A	120 (2)	N6C—C3C—N3C	121.51 (18)
C3A—N6A—H5A	120.8 (16)	N6C—C3C—N1C	116.79 (18)
C3A—N6A—H6A	117.5 (14)	N3C—C3C—N1C	121.70 (17)
H5A—N6A—H6A	122 (2)	C3D—N1D—C1D	114.55 (16)
N4A—C1A—N2A	120.93 (18)	C1D—N2D—C2D	114.67 (16)
N4A—C1A—N1A	117.61 (18)	C3D—N3D—C2D	114.38 (16)
N2A—C1A—N1A	121.47 (17)	C1D—N4D—H1D	119.3 (13)
N5A—C2A—N3A	116.94 (17)	C1D—N4D—H2D	116.6 (15)
N5A—C2A—N2A	116.77 (18)	H1D—N4D—H2D	123 (2)
N3A—C2A—N2A	126.28 (18)	C2D—N5D—H3D	117.8 (15)
N6A—C3A—N3A	121.21 (18)	C2D—N5D—H4D	115.0 (16)
N6A—C3A—N1A	117.70 (18)	H3D—N5D—H4D	126 (2)
N3A—C3A—N1A	121.08 (17)	C3D—N6D—H5D	118.3 (14)
C1B—N1B—C3B	114.54 (16)	C3D—N6D—H6D	115.0 (14)
C1B—N2B—C2B	114.46 (16)	H5D—N6D—H6D	120 (2)
C2B—N3B—C3B	115.23 (16)	N4D—C1D—N2D	117.56 (18)
C1B—N4B—H1B	116.4 (14)	N4D—C1D—N1D	117.41 (18)
C1B—N4B—H2B	120.1 (14)	N2D—C1D—N1D	125.02 (18)
H1B—N4B—H2B	121 (2)	N5D—C2D—N2D	117.53 (18)
C2B—N5B—H3B	118.4 (15)	N5D—C2D—N3D	116.89 (18)
C2B—N5B—H4B	117.1 (15)	N2D—C2D—N3D	125.58 (17)
H3B—N5B—H4B	122 (2)	N3D—C3D—N1D	125.78 (17)
C3B—N6B—H5B	122.0 (15)	N3D—C3D—N6D	118.13 (18)
C3B—N6B—H6B	118.3 (14)	N1D—C3D—N6D	116.07 (17)
H5B—N6B—H6B	120 (2)	O3AB—N8A—O2AA	169.5 (9)
N4B—C1B—N1B	117.02 (18)	O3AB—N8A—O1AA	57.0 (9)
N4B—C1B—N2B	117.46 (17)	O2AA—N8A—O1AA	120.86 (17)
N1B—C1B—N2B	125.51 (17)	O3AB—N8A—O1AB	129.1 (12)
N5B—C2B—N3B	118.21 (17)	O2AA—N8A—O1AB	52.2 (7)
N5B—C2B—N2B	116.93 (17)	O1AA—N8A—O1AB	73.0 (8)
N3B—C2B—N2B	124.86 (17)	O3AB—N8A—O3AA	63.9 (9)
N6B—C3B—N3B	117.65 (18)	O2AA—N8A—O3AA	119.80 (17)
N6B—C3B—N1B	117.06 (18)	O1AA—N8A—O3AA	119.28 (16)
N3B—C3B—N1B	125.28 (17)	O1AB—N8A—O3AA	154.5 (8)
C1C—N1C—C3C	119.51 (17)	O3AB—N8A—O2AB	118.6 (12)
C1C—N1C—H2N	120.2 (15)	O2AA—N8A—O2AB	58.6 (7)
C3C—N1C—H2N	120.2 (14)	O1AA—N8A—O2AB	156.4 (7)
C1C—N2C—C2C	115.68 (16)	O1AB—N8A—O2AB	110.8 (11)
C3C—N3C—C2C	115.38 (17)	O3AA—N8A—O2AB	67.8 (7)
C1C—N4C—H1C	118.9 (15)	O2B—N8B—O1B	120.62 (16)
C1C—N4C—H2C	122.7 (15)	O2B—N8B—O3B	120.05 (16)
H1C—N4C—H2C	118 (2)	O1B—N8B—O3B	119.34 (16)
C2C—N5C—H3C	117.8 (14)	H1W1—O1W—H2W1	102 (3)
C2C—N5C—H4C	119.4 (15)	H1W2—O2W—H2W2	104 (2)
			
C2A—N2A—C1A—N4A	−179.15 (18)	C2C—N2C—C1C—N4C	−179.32 (18)
C2A—N2A—C1A—N1A	0.9 (3)	C2C—N2C—C1C—N1C	0.9 (3)
C3A—N1A—C1A—N4A	−178.41 (18)	C3C—N1C—C1C—N4C	−177.41 (18)
C3A—N1A—C1A—N2A	1.5 (3)	C3C—N1C—C1C—N2C	2.4 (3)
C3A—N3A—C2A—N5A	−178.58 (18)	C1C—N2C—C2C—N5C	176.16 (18)
C3A—N3A—C2A—N2A	2.8 (3)	C1C—N2C—C2C—N3C	−4.2 (3)
C1A—N2A—C2A—N5A	178.11 (18)	C3C—N3C—C2C—N5C	−176.55 (18)
C1A—N2A—C2A—N3A	−3.2 (3)	C3C—N3C—C2C—N2C	3.8 (3)
C2A—N3A—C3A—N6A	179.66 (19)	C2C—N3C—C3C—N6C	−179.85 (19)
C2A—N3A—C3A—N1A	0.0 (3)	C2C—N3C—C3C—N1C	−0.1 (3)
C1A—N1A—C3A—N6A	178.31 (18)	C1C—N1C—C3C—N6C	176.93 (18)
C1A—N1A—C3A—N3A	−2.0 (3)	C1C—N1C—C3C—N3C	−2.8 (3)
C3B—N1B—C1B—N4B	−176.03 (18)	C2D—N2D—C1D—N4D	179.39 (17)
C3B—N1B—C1B—N2B	2.6 (3)	C2D—N2D—C1D—N1D	0.3 (3)
C2B—N2B—C1B—N4B	178.92 (18)	C3D—N1D—C1D—N4D	−179.25 (17)
C2B—N2B—C1B—N1B	0.3 (3)	C3D—N1D—C1D—N2D	−0.2 (3)
C3B—N3B—C2B—N5B	−178.07 (17)	C1D—N2D—C2D—N5D	178.84 (17)
C3B—N3B—C2B—N2B	2.4 (3)	C1D—N2D—C2D—N3D	−1.2 (3)
C1B—N2B—C2B—N5B	177.45 (17)	C3D—N3D—C2D—N5D	−178.23 (17)
C1B—N2B—C2B—N3B	−3.0 (3)	C3D—N3D—C2D—N2D	1.8 (3)
C2B—N3B—C3B—N6B	−179.74 (18)	C2D—N3D—C3D—N1D	−1.7 (3)
C2B—N3B—C3B—N1B	1.0 (3)	C2D—N3D—C3D—N6D	179.96 (17)
C1B—N1B—C3B—N6B	177.44 (18)	C1D—N1D—C3D—N3D	0.9 (3)
C1B—N1B—C3B—N3B	−3.3 (3)	C1D—N1D—C3D—N6D	179.31 (17)

### Hydrogen-bond geometry (Å, °)

**Table:**

*D*—H···*A*	*D*—H	H···*A*	*D*···*A*	*D*—H···*A*
N4A—H1A···O2W^i^	0.92 (2)	2.05 (2)	2.965 (2)	174.2 (17)
N4B—H1B···N1D	0.85 (2)	2.18 (2)	3.025 (2)	174.1 (18)
N4D—H1D···O3AA	0.88 (2)	2.27 (2)	3.077 (2)	153 (2)
N1A—H1N···O1W^ii^	0.91 (2)	1.89 (2)	2.771 (2)	165 (2)
N4A—H2A···O1B^i^	0.96 (3)	2.03 (3)	2.838 (2)	141 (2)
N4B—H2B···O3AA	0.93 (3)	2.05 (2)	2.810 (2)	138.8 (18)
N4C—H2C···N2D^iii^	0.88 (3)	2.07 (3)	2.945 (2)	176 (2)
N4D—H2D···N2C^iii^	0.84 (2)	2.23 (2)	3.069 (2)	172 (2)
N1C—H2N···O3B	0.93 (2)	1.91 (2)	2.836 (2)	177 (2)
O2W—H1W2···N1D	0.86 (3)	2.04 (3)	2.899 (2)	174 (3)
N5A—H3A···N2B	0.88 (3)	2.20 (3)	3.062 (2)	165 (2)
N5B—H3B···N2A	0.82 (3)	2.30 (3)	3.119 (2)	175 (2)
N5C—H3C···O2AA^iii^	0.89 (2)	2.06 (2)	2.799 (2)	140 (2)
N5D—H3D···O3AA^iv^	0.86 (2)	2.53 (2)	3.232 (3)	140 (2)
N5A—H4A···O1AA	0.86 (2)	2.11 (2)	2.963 (2)	171 (2)
N5B—H4B···O2B^i^	0.86 (2)	2.20 (2)	3.045 (2)	167 (2)
N5C—H4C···N3D^v^	0.89 (3)	2.12 (3)	2.996 (2)	173 (2)
N5D—H4D···N3C^v^	0.81 (3)	2.30 (3)	3.105 (3)	173 (2)
N6A—H5A···N3B^vi^	0.80 (2)	2.13 (2)	2.926 (2)	175.9 (19)
N6B—H5B···N3A^vii^	0.84 (2)	2.18 (2)	3.020 (2)	177 (2)
N6C—H5C···O2W	0.93 (3)	2.00 (3)	2.849 (2)	151 (2)
N6D—H5D···O2AA^vii^	0.93 (3)	2.22 (3)	3.126 (2)	167 (2)
N6A—H6A···O1W^ii^	0.90 (2)	2.55 (2)	3.262 (2)	137.2 (15)
N6A—H6A···O2B^viii^	0.90 (2)	2.14 (2)	2.841 (2)	135.2 (17)
N6B—H6B···O1AA^vii^	0.89 (2)	2.17 (3)	2.806 (2)	128 (2)
N6C—H6C···O1B	0.90 (2)	1.97 (2)	2.868 (2)	174.6 (18)
N6D—H6D···N1B	0.91 (2)	2.18 (2)	3.088 (2)	172 (2)

Symmetry codes: (i) −*x*+1, −*y*+1, −*z*; (ii) *x*+1, *y*, *z*−1; (iii) −*x*+1, −*y*+2, −*z*+1; (iv) −*x*+1, −*y*+1, −*z*+1; (v) −*x*, −*y*+1, −*z*+1; (vi) *x*+1, *y*+1, *z*; (vii) *x*−1, *y*−1, *z*; (viii) −*x*+2, −*y*+2, −*z*.
