# Melaminium nitrate–melamine–water (1/1/1). Corrigendum

**DOI:** 10.1107/S1600536810047847

**Published:** 2010-11-23

**Authors:** Farook Adam, Kei Lin Sek, Kasim Mohammed Hello, Madhukar Hemamalini, Hoong-Kun Fun

**Affiliations:** a School of Chemical Sciences, Universiti Sains Malaysia, 11800 USM, Penang, Malaysia; b School of Chemistry, Universiti Sains Malaysia, 11800 USM, Penang, Malaysia; c X-ray Crystallography Unit, School of Physics, Universiti Sains Malaysia, 11800 USM, Penang, Malaysia

## Abstract

The name of one of the authors in the paper by Adam *et al.* [*Acta Cryst.* (2010), E**66**, o3033–o3034] is corrected.

## Abstract 2

Corrigendum to *Acta Cryst.* (2010), E**66**, o3033–o3034.

In the paper by Adam *et al.* (2010) ▶, the second author is incorrectly given as ‘Sek Kei Lin’. The correct name should be ‘Kei Lin Sek’, as given above.
